# Soluble T-Cell Receptors Produced in Human Cells for Targeted Delivery

**DOI:** 10.1371/journal.pone.0119559

**Published:** 2015-04-13

**Authors:** Even Walseng, Sébastien Wälchli, Lars-Egil Fallang, Weiwen Yang, Anette Vefferstad, Ali Areffard, Johanna Olweus

**Affiliations:** 1 Department of Immunology, Institute for Cancer Research, Oslo University Hospital Radiumhospitalet, Oslo, Norway; 2 Department of Cell Therapy, Institute for Cancer Research, Oslo University Hospital Radiumhospitalet, Oslo, Norway; 3 K. G. Jebsen Center for Cancer Immunotherapy and K.G. Jebsen Center for Inflammation Research, Institute for Clinical Medicine, University of Oslo, Oslo, Norway; University of London, St George's, UNITED KINGDOM

## Abstract

Recently, technology has become available to generate soluble T-cell receptors (sTCRs) that contain the antigen recognition part. In contrast to antibodies, sTCRs recognize intracellular in addition to extracellular epitopes, potentially increasing the number of applications as reagents for target detection and immunotherapy. Moreover, recent data show that they can be used for identification of their natural peptide ligands in disease. Here we describe a new and simplified expression method for sTCRs in human cells and show that these sTCRs can be used for antigen-specific labeling and elimination of human target cells. Four different TCRs were solubilized by expression of constructs encoding the TCR alpha (α) and beta (β) chains lacking the transmembrane and intracellular domains, linked by a ribosomal skipping 2A sequence that facilitates equimolar production of the chains. Cell supernatants containing sTCRs labeled target cells directly in a peptide (p)-human leukocyte antigen (HLA)-specific manner. We demonstrated that a MART-1p/HLA-A*02:01-specific sTCR fused to a fluorescent protein, or multimerized onto magnetic nanoparticles, could be internalized. Moreover, we showed that this sTCR and two sTCRs recognizing CD20p/HLA-A*02:01 could mediate selective elimination of target cells expressing the relevant pHLA complex when tetramerized to streptavidin-conjugated toxin, demonstrating the potential for specific delivery of cargo. This simple and efficient method can be utilized to generate a wide range of minimally modified sTCRs from the naturally occurring TCR repertoire for antigen-specific detection and targeting.

## Introduction

While T-cell receptors (TCRs) are transmembrane proteins and do not naturally exist in soluble form, antibodies can be secreted as well as membrane bound. Importantly, TCRs have the advantage over antibodies that they in principle can recognize peptides generated from all degraded cellular proteins, both intra- and extracellular, when presented in the context of MHC molecules. Thus, the number of potential TCR targets vastly exceeds that of antibodies.

Soon after the characterization of the TCR structure [1], attempts to create soluble TCRs (sTCRs) were undertaken [2]. Currently, bacteria constitute the main platform for sTCR production, although *Drosophila* cells have also proven efficient [3]. The two TCR chains are expressed separately, isolated in inclusion bodies and refolded together (reviewed in [4–6]), but the TCR chains produced by this approach appear to be very unstable. The yield was, however, improved when the membrane proximal cysteines forming the interchain disulfide bridge were excluded [7], or when a cysteine bridge was added to the constant domains [8]. Nevertheless, the success rate for correct refolding was recently estimated to be as low as 33% [9], presenting the need for a more efficient production system. Initially, sTCRs were mainly produced for crystallography purposes, as reviewed in [10], but were also used to characterize specificity and affinity in conjunction with repertoire analysis [5], and for substrate identification [11, 12]. Results from such studies facilitated the design of full-length TCRs for re-direction of T cells in adoptive cellular therapy settings [13–15]. Soluble TCRs intended for therapeutic use have been produced, including modified versions designed as single-chain proteins [16–20]. This design is limited by the linker sequence that artificially connects the V regions, potentially resulting in reduced or altered antigen recognition and in immunogenicity.

In the present report we describe a novel approach for the production and delivery of sTCRs. Human cells were chosen as the platform of production for two reasons. First, it permits the use of bicistronic vectors encoding both the TCRα and β chains separated by the ribosomal skipping sequence 2A found in the picorna virus [21]. This facilitates equimolar production of the alpha and the beta chains and efficient folding, as previously shown for the full-length TCR/CD3 complex [22]. Second, production in mammalian cells allows post-translational modifications. The resulting sTCRs are thus close to their natural counterparts, which reduces immunogenicity and might increase therapeutic potential, as well as applicability in recently developed assays for identification of natural TCR-ligands that represent the driving antigen in autoimmune disease [23], or curative immune responses to cancer. The construct was designed to remain as close to the full-length counterpart as possible, only removing the transmembrane and intracellular domains. Here, we demonstrate that non-affinity matured sTCRs produced by a simple and efficient method in human cells can detect, be internalized into and deliver cytotoxic cargo to target cells expressing the cognate pHLA complex.

## Materials and Methods

### Antibodies and peptides

The following antibodies were used: anti-CD3 (OKT-3) (BD Biosciences, Erembodegem, Belgium), anti-FLAG (M2) (Sigma-Aldrich St. Louis, MO, USA), anti-His (MCA1396) (AbD Serotec, Kidlington, Oxford, UK), anti-HLA-A2 (BB7.2; AbD Serotec) and goat anti-mouse Phycoerythrin (PE) (Jackson ImmunoResearch, PA, USA). All peptides were synthesized by GenScript (Piscataway, NJ, USA): MART-1 peptide_26–35_ (ELAGIGILTV), CD20 peptide_188–196_ (SLFLGILSV) and EBV peptide_280–288_ (GLCTLVAML). AF647-labeled antibodies were prepared in-house using a protein labeling kit from Life Technologies (Grand Island, NY, USA).

### Cell culture

SupT1 cells (ECACC, 95013123) were a kind gift from M. Pule (University College London, UK) and Platinum-E cells (HEK293T derivative, referred to as HEK293) were from Cell Biolabs (San Diego, CA, USA). HeLa cells were a kind gift from the Department of Biochemistry, Institute for Cancer Research (Oslo University Hospital, Norway). HEK293 and HeLa were grown in DMEM (PAA Laboratories GmbH, Germany). All media were supplemented with 10% heat-inactivated fetal calf serum (FCS, HyClone, Logan, UT, USA) and 100 U/mL penicillin/streptomycin (PAA Laboratories GmbH, Germany).

### Cloning

The sTCR constructs were cloned using a 2-step procedure similar to the one described previously [24]. Since we possessed all the constructs as full length TCR connected to a 2A peptide, we used a V α specific primers, as in [24] and internal primers removing the transmembrane region and cytoplasmic tail. Since the 2A sequence the V β could be amplified through the 2A without the need of using a V β-specific primer, we designed universal primers which would create a anchoring sequence on the 2A but could be modified to adapt a tag at the 3’-end of the α-chain. The reverse primer of the TCR α (extracellular-tag-2A) was annealed with the 2A forward (V βamplification) in the second PCR round. Thus, the internal primer sequences are: 5’-ctcttttggctctacaggaactttctgggctg-3’ and 5’-cagaaagttcctgtagagccaaaagagagggcag-3’. The TCR β reverse primer for His-tagging: 5’-attctcgagttactagtgatggtgatggtgatgacctccaccacagtctgctctaccccag-3’. The FLAG-tag on DMF5 sTCR (alpha-chain) was added by site directed mutagenesis (SDM) using these primers: 5’-cccagaaagttcctgtggaggtggtgactacaaggacgacgacgacaagagagccaaaagagagg-3’ and 5’-cctctcttttggctctcttgtcgtcgtcgtccttgtagtcaccacctccacaggaactttctggg-3’. The addition of the BirA sequence was performed by amplifying any sTCR with a specific forward primer and the following reverse primer: 5’-ttc tcg agt cag tgc cac tcg atc ttc tgg gcc tcg aag atg tcg ttc agg ccg cca cca cag tct gct cta ccc cag-3’. Furthermore, we re-inserted a His-tag between the TCR β-chain and the BirA tag by SDM using these primers: 5’-ggggtagagcagactgtggaggaggacatcaccatcaccatcacggtggcggcc tgaacgac-3’ and 5’-gtcgttcaggccgccaccgtgatggtgatggtgatgtcctcctccacagtctgc tctacccc-3’. For the mCherry fusion construct, we used mCherry cDNA as template and amplified it with the following primers 5’-agcagactgtggtggaggtatggtgagcaag-3’ and 5’- ctcgagtcagtgccactcgatcttctgggcctcgaagatgtcgttcaggccgccacccttgtacagctcgtccatgc (including BirA tag) or 5’- to generate an mCherry-BirA fragment. This product was combined with the sTCR containing a FLAG sequence on the α-chain. We finally re-introduced the His-tag between mCherry and BirA by SDM using the following primers: 5’-ggacgagctgtacaagggaggaggacatcaccatcaccatcacggtggcggcctgaacgac and 5’- gtcgttcaggccgccaccgtgatggtgatggtgatgtcctcctcccttgtacagctcgtcc-3’.

To increase the interchain stability of certain sTCRs, a high affinity leucine zipper (LZ) pair following a flexible (G_4_S)_4_ linker was attached to the C terminus of the chains through sewing PCR. This design was based on work by Moll et al [25]. In addition, a FLAG-tag (Sigma-Aldrich) was added to the α-chain for isolation and detection. The LZ sequences were purchased from Genscript: 5’-ctggaaattcgcgcggcgtttctgcgccagcgcaacaccgcgctgcgcaccgaagtggcgga actggaacaggaagtgcagcgcctggaaaacgaagtgagccagtatgaaacccgctatggcccgctg-3’ and 5’- ctggaaattgaagcggcgtttctggaacgcgaaaacaccgcgctggaaacccgcgtggcggaactgcgccagcgcgtgcagcgcctgcgcaaccgcgtgagccagtatcgcacccgctatggcccgctg-3’. HLA-A2-peptide SCT constructs were based on the murine MHC equivalent, explained in [26]. Briefly, the synthesized gene (Eurofins MWG Operon, Ebersberg, Germany) consisted of the antigenic peptide, β2m and heavy chain of HLA-A2 linked by means of 15 and 20 aa G4S linkers, respectively. In addition, cysteines were introduced in position 84 of the heavy chain and in the peptide-β2m linker to ensure the opening of the peptide-binding groove, and to create a disulfide trap for increased binding of the peptide in the groove. To simplify the peptide exchange, *Xho*I and *Afe*I restriction enzyme sites flanked the peptide sequence. The gene was cloned into the Gateway compatible vector pENTR-D (Invitrogen) for easy cloning into the Gateway compatible pMP71 retroviral expression vector. Retrovirus was produced as previously published [24], and HLA-A2 negative (HLA-A2^neg^) SupT1 cells were transduced and subsequently FACS sorted based on HLA-A2 expression. The HLA-A2 construct was previously described in [27] and was used as a template for site directed mutagenesis to create a DK mutant using the following primers: HLA_DTKA_F 5’-gaggaccagacccagaaggcggagctcgtggagac-3’ and HLA_DTKA_R 5’-gtctccacgagctccgc cttctgggtctggtcctc-3’.

### Soluble protein expression, purification and Western blot

For production of the sTCR, HEK293 were transfected using XtremeGene 9 (Roche, Basel, Switzerland) as recommended by the manufacturer, and the supernatants were collected 72 h post-transfection. The sTCR was purified by immunoprecipitation by passing the supernatant over an M2-sepharose column (Sigma-Aldrich) and eluted with FLAG peptide as recommended by the manufacturer (Sigma-Aldrich). The eluate was concentrated using a Vivaspin ultrafiltration spin column with 30.000 MWCO (Sartorius AG, Göttingen, Germany). For immune blotting, supernatants from cells expressing sTCR or not were boiled in loading buffer with or w/o β-mercaptoethanol (5% final concentration in reducing condition) (Thermo Fisher Scientific Inc., Rockford, IL USA) and subjected to SDS-PAGE followed by transfer to a nitrocellulose membrane and labeling with the primary abs described in the figure legend and subsequent staining with HRP-conjugated anti-mouse IgG (Southern Biotechnology Associates, Birmingham, AL).

### Biotinylation and multimerization

Biotinylation of the BirA sequence containing sTCR proteins was performed as recommended by the manufacturer (Avidity, Aurora, CO, USA). Multimerization was completed by incubating 235 nM sTCR with 45 nM of SA-PE (Life Technologies) for 40 minutes at RT. To produce TCR conjugated to saporin (sTCR-Sap) we purchased Streptavidin-ZAP (Advanced Targeting Systems, San Diego, CA, USA) and followed the manufacturer′s recommendation to obtain a stock of sTCR-Sap of 180 nM.

### Staining of cells for confocal microscopy and flow cytometry

HeLa cells seeded out on cover slips were transfected with CD20-mCherry and SCT-M1-mCherry using Xtremegene-9 (Roche) according to the manufacturer’s protocol. The cells were fixed in 4% paraformaldehyde in PBS at RT and subsequently stained with mouse anti-His AF488 (Life Technologies) in PBS containing 0.1% saponin and mounted on slides. SupT1 cells transfected with SCT-M1 were fastened to Poly-L-lysine (Sigma) treated cover slips and subsequently incubated with DMF5 sTCR-mCherry for 30 minutes at 37°C. The cells were then stained with mouse anti-His on ice, and finally stained with goat anti-mouse AF488 (Molecular Probes), fixed with 4% PFA and mounted on slides. sTCR-mCherry, SA-Dynabeads (Life Technologies) or SA-Miltenyi nanobeads (Miltenyi, Bergisch Gladbach, Germany) were incubated with cells for 45 minutes at 37°C. Cells were then attached to Poly-L-lysine treated cover slips and mounted to slides. All cells were imaged utilizing a Zeiss LSM 710 confocal microscope using a ×63 oil immersion objective lens (NA 1.4). Staining for flow cytometric detection of sTCR binding was performed as follows: 10^5^ cells were incubated in 20 μl with sTCR for 15 min at RT, where indicated subsequent to pulsing with indicated concentrations of peptide overnight. After washing they were stained with anti-His AF647 or anti-FLAG AF647 for an additional 15 min at RT before flow cytometric analysis on an LSRII flow cytometer (BD Biosciences, San José, CA, USA). For staining with nanobeads, 10^5^ peptide-loaded SupT1 cells were washed and 2.25 μL beads were added for 5min at 37°C. SA-beads (Miltenyi) pre-incubated with biotinylated CD20 swap sTCR monomer were used. Cells were washed and stained with anti-FLAG AF647 for 15 min at RT.

### Assays to measure effects of toxin-conjugated sTCR

Target cells were incubated in complete medium at 10^5^ cells in 200 μl/well in a 96-well plate. A preparation of sTCR-Sap was added to a final concentration of 10 or 20 nM and cultures were left for 3 days at 37 ºC. For the ^3^H-thymidin incorporation assay, cell proliferation was measured after labeling with 3.7x10^4^ Bq [^3^H] thymidine (Laborel, Oslo, Norway) for 4 h at 37ºC. For flow cytometry-based measurement of elimination of target cells, harvested cells were stained with anti-HLA-A2 AF647 for flow cytometric measurements of frequencies of HLA-A2 positive and negative targets cells in each well. Percentage eliminated target cells was determined as mean of duplicates of 1-(%HLA-A2 pos cells with sTCR / %HLA-A2 pos cells w/o sTCR).

## Results

### Production of 2A peptide-linked soluble TCRs in human cells

**Fig 1:**
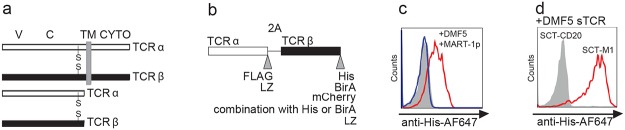
Design of the sTCR and its use as a labeling reagent. (a) TCRα/β chains in full-length (upper part) and in soluble form (lower part). V: Variable domain, C: Constant domain, TM: transmembrane, CYTO: cytosolic domain. Interchain cystein bridges are indicated with S. (b) Design of the expression construct in which truncated TCRα and β chains are separated by a ribosome skipping sequence (2A) and tagged on their 3’-end. The tags used in this study are listed at their respective position. To increase interchain stability, a high affinity leucine zipper (LZ) was added to some sTCR constructs. (c) HLA-A2^pos^ T2 cells were loaded O/N with 10 μM MART-1p (red, blue) or 10 μM of an irrelevant peptide (CD20p_188–196_) (filled grey). Ten μL of supernatant from HEK293 cells producing the DMF5 sTCR (red), or from mock transfected HEK 293 (blue), was added to the T2 cells and incubated for 15 minutes at RT followed by labeling with anti-His-647 antibodies and flow cytometric analysis (d) SupT1 cells expressing SCT-M1 (red) or SCT-CD20 (filled grey) were incubated with the DMF5 sTCR supernatant as described in (c) and stained using anti-His-647. The results in (c) and (d) are representative of at least two experiments and histograms are gated on viable cells, displayed as FSC^hi^, SSC^hi^ events.

We first tested the possibility of producing sTCRs by removing the transmembrane and cytosolic parts of the alpha and beta chains (Fig 1a) and linking them together by a Picorna virus 2A sequence (Fig 1b) using the TCR DMF5 [28]. It recognizes the MART-1 peptide_27–35_ (EAAGIGILTV, referred to as WT MART-1p) [29], and with higher affinity the improved peptide ELAGIGILTV (referred to as MART-1p), in complex with HLA-A*02:01. This TCR was selected because it shows partial CD8-independency [30], and has been previously produced in bacteria [31]. The first DMF5 sTCR variant was His-tagged on the C-terminal end of the TCRβ chain while the α-chain was kept unmodified (Fig 1b). The HEK293 cells were transfected with the construct, and the supernatant specifically bound HLA-A2^pos^ T2 cells loaded with MART-1p (Fig 1c). Moreover, the DMF5 sTCR bound to HLA-A2^neg^ cells transduced to constitutively express an MHC class I single-chain trimer (SCT) [32] containing the MART-1p (SCT-M1) (Fig 1d). The induced expression of both SCTs resulted in a strong signal when staining with anti-HLA-A2 antibodies (S1 Fig). Taken together, these data indicated that the DMF5 sTCR was produced and released into the medium as a functional molecule.

**Fig 2:**
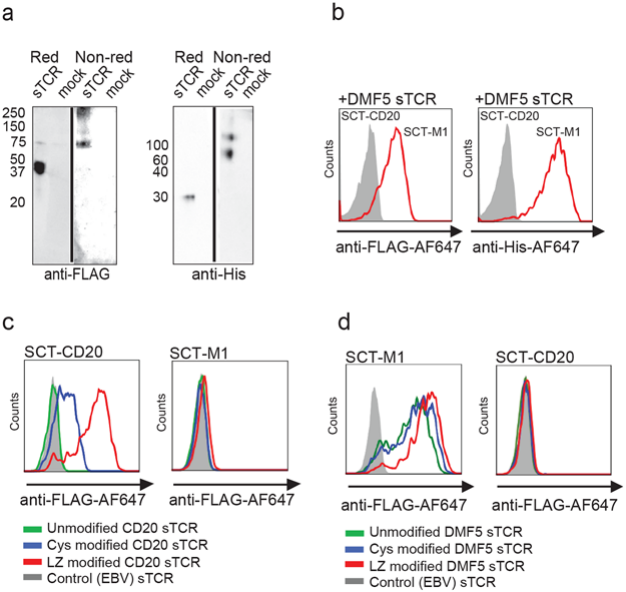
The sTCR is produced as a heterodimer and recognizes the correct pMHC. (a) Supernatant from HEK293 cells expressing FLAG- and His-tagged DMF5 sTCR or not (mock) was loaded on SDS-PAGE under reducing (Red) or non-reducing (Non-red) conditions. The membrane was subsequently stained with anti-His or anti-FLAG antibodies. (b) The same sTCR as in (a) was used to stain SupT1 cells expressing SCT-M1 (red) or SCT-CD20 (filled grey) and stained with anti-FLAG-647 (left panel) or anti-His-AF647 (right panel). The results in (a) and (b) are representative of at least 2 experiments. (c) SupT1 cells expressing SCT-CD20 (left panel) or SCT-M1 (right panel, negative control) were incubated with unmodified (green), cys modified (blue) or sTCR LZ modified (red) CD20 swap-sTCR. They were visualized using anti-FLAG-647 antibody. EBV-sTCR (filled grey) served as a negative control. (d) SupT1 cells expressing SCT-M1 (left panel) or SCT-CD20 (right panel, negative control) were incubated with unmodified DMF5 sTCR (green), DMF5 sTCR with a cys modification (blue) or DMF5 sTCR LZ (red), and subsequently visualized using an anti-FLAG-647 antibody. As a negative control, the SCT-M1 cells were also incubated with an irrelevant EBV-sTCR (filled grey). In (b-d), histograms are gated on viable cells, displayed as FSC^hi^, SSC^hi^ events.

To verify that the sTCR was indeed a heterodimer, we designed a double tagged construct of the DMF5 sTCR (Fig 1b). This construct was expressed and the cell supernatant separated on SDS-PAGE under reducing or non-reducing conditions (Fig 2a). Proteins were detected by Western blotting, and a protein migrating around 30 kDa was observed in reducing conditions (expected size of a single TCR chain) (Fig 2a). An additional band of double molecular weight (MW) was detected in non-reducing conditions, suggesting that the sTCR was expressed as a heterodimeric molecule. Furthermore, this dimer was sensitive to the reducing agent, indicating a disulfide bond linkage. We also observed signals for both antibodies at higher MW in non-reducing conditions, possibly representing multimers, which could explain the surprisingly efficient staining of the supernatant, in which the sTCRs were not multimerized by streptavidin. This double-tagged sTCR was subsequently used to stain SCT-M1 expressing cells, and binding could be detected using either anti-FLAG or anti-His antibodies, supporting the presence of the two chains in a functional heterodimer (Fig 2b). Collectively, these results demonstrated that a specific and functional dimeric sTCR transcribed from a single coding sequence can be produced in human cells.

Next, we investigated if the technology could be utilized for the production of a TCR that has not previously been made soluble, and which contains α- and β-chains that do not naturally pair well. To this end, we selected a TCR sequence composed of an α and a β chain derived from either of two naturally occurring TCRs, each recognizing a peptide from the B-cell specific protein CD20 presented on allogeneic HLA-A*02:01 [33]. This α- and β-chain combination did not produce a functional sTCR dimer. The chains were thus modified to express either a cysteine bridge (cys) or a leucine zipper (LZ) pair [3, 34–36] (Fig 1b). The supernatant containing the CD20 swap-sTCR-cys, and to an even higher degree the CD20 swap-sTCR-LZ, labeled target cells expressing SCT-CD20, but not cells expressing a control SCT-M1 (Fig 2c), indicating that a functional sTCR was generated. To assess whether or not the LZ modification alters the specificity of the TCR, the zipper pair was added to the high affinity DMF5 sTCR, which retained specific binding to the SCT-M1 (Fig 2d). Thus, the interchain affinity can be increased by addition of LZ to increase the production of soluble TCRs also from α and β chains that do not naturally pair well.

### Multimerization of the sTCR increases the sensitivity of target detection

**Fig 3:**
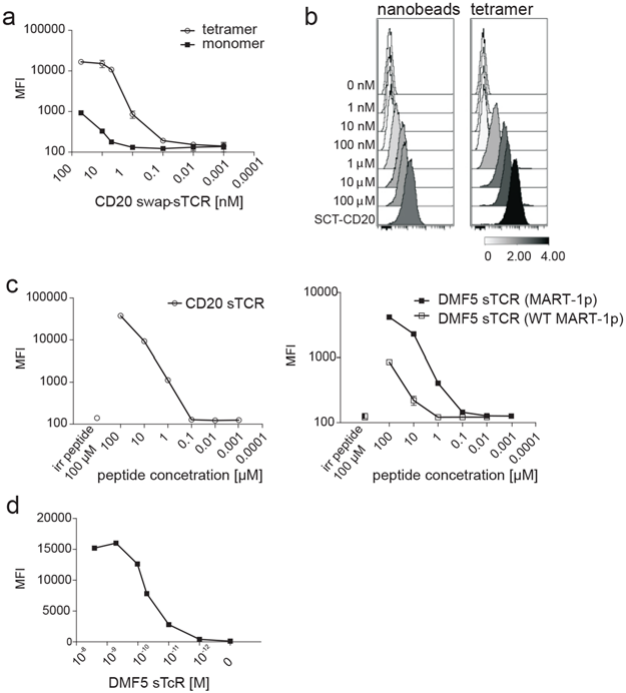
Multimerization of the sTCR increases sensitivity of antigen detection by flow cytometry. (a) SupT1 cells expressing SCT-CD20 were incubated with increasing concentrations of CD20 swap-sTCR monomer or tetramer, as indicated, and indirectly labeled with anti-FLAG AF647. Results shown are representative of two experiments, and error bars represent SD of duplicates (b) HLA-A2^+^ SupT1 cells were loaded with indicated concentrations of peptide and subsequently labeled with saturating amounts of either sTCR tetramer or nanobeads conjugated to sTCR monomers, followed by staining with anti-FLAG-AF647. Results shown are representative of two experiments. SupT1 cells expressing SCT-CD20 were used as a positive control. The scale shows the calculation of Arcsinh ratio of the Median. (c) SupT1 cells were loaded with increasing concentrations of CD20p (left panel), MART-1p or WT MART-1p (right panel), followed by labeling with saturating amounts of indicated sTCR tetramer, and indirectly stained with anti-FLAG AF647. Irrelevant peptide control (irr peptide) was used at 100 μM (disconnected symbols). Results shown are from one experiment representative of 3 (CD20p and MART-1p) or 2 (WT MART-1p) experiments. Error bars indicate SD of duplicates. (d) SupT1 cells expressing SCT-M1 were incubated with increasing concentrations of PE-conjugated sTCR tetramer.

We next explored the possibility of increasing the sensitivity of staining by multimerizing purified sTCR. A BirA sequence (GGLNDIFEAQKIEWH) was introduced at the end of the TCR β-chain for subsequent biotinylation (Fig 1b). The CD20 swap-sTCR was purified, biotinylated, and tetramerized to streptavidin conjugated to phycoerythrin (SA-PE). Tetramers displayed a higher sensitivity for antigen-detection than monomers (Fig 3a). Also sTCR multimerized onto magnetic nanobeads and showed that similar levels of staining could be achieved as for tetramers, expanding the possibilities for our technology to include cell purification (Fig 3b).

The sensitivity of antigen-detection was determined to be at least 1 μM for recognition of the CD20p by the CD20 swap-sTCR and the MART-1p by the DMF5 sTCR. The WT MART-1p was recognized at 10 μM by the DMF5 (Fig 3c), likely reflecting the lower affinity of this peptide for HLA-A2. Target cells loaded with high concentrations (100 μM) of irrelevant peptide were not recognized, showing specificity (Fig 3c). Finally, since SCT represents a situation in which all the MHC molecules are loaded with the same peptide, we could determine that the EC_50_ of DMF5 SA-PE sTCR binding to its target was 0.1 nM (Fig 3d).

Collectively, these data show that sTCR produced in human cells can accommodate a variety of modifications, including fluorescent proteins or tags for labeling and multimerization, increasing the sensitivity with which the cognate pHLA complex can be detected and the possibilities for additional applications, such as cell isolation.

### Soluble TCRs are internalized upon binding of the cognate pHLA ligand

**Fig 4:**
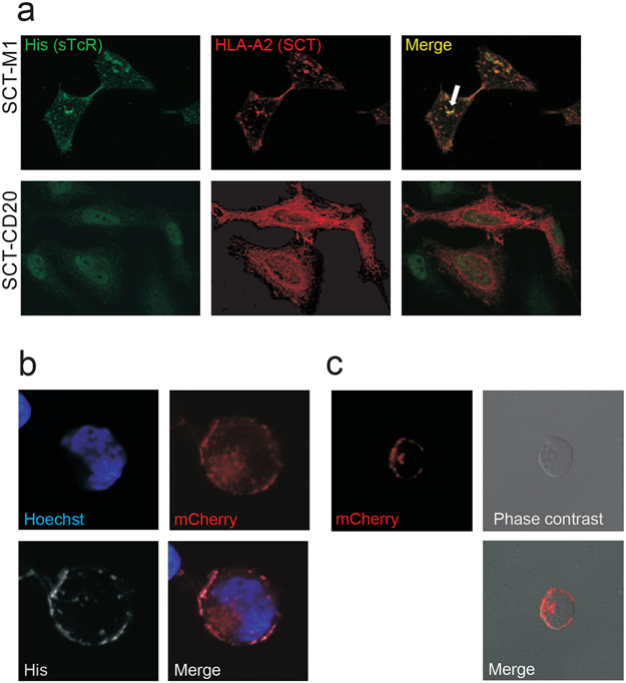
DMF5 sTCR is internalized upon specific ligand binding. (a) HeLa cells transfected with either SCT-M1-mCherry or SCT-CD20-mCherry (red) were incubated with His-tagged DMF5 sTCR labeled with an anti-His antibody and visualized using anti-mouse AF488 (green). Co-localization of the sTCR and SCT-M1 is shown in yellow (arrow). (b) Sup-T1 cells expressing SCT-M1 were incubated with DMF5 supernatants containing monomeric sTCR mCherry-His at 37°C for 30 minutes. The cells were subsequently put on ice to block endocytosis and stained with anti-His-AF647 (His). The nucleus was visualized by Hoechst stain (blue). (c) Sup-T1 cells expressing SCT-M1 were incubated with biotinylated DMF5 sTCR-mCherry bound to SA-Miltenyi nanobeads at 37°C.

Peptide-MHC complexes are constitutively internalized and recycled [37]. We therefore tested if this mechanism could be utilized to transport sTCRs inside the cell. HLA-A2^neg^ HeLa cells were transfected with SCT-M1 showed surface staining with the MART-1 sTCR, and merged staining of the SCT-M1 and the sTCR suggested that the two proteins interacted and co-localized within the cell (Fig 4a). The sTCR was unable to stain HeLa cells expressing SCT-CD20, demonstrating specificity.

Next, we examined to which extent the sTCR was capable of carrying cargo into the cell. To this end, a DMF5 sTCR was designed in which the β-chain was tagged with the fluorescent protein mCherry followed by a His-tag (mCherry-His-sTCR, Fig 1b). HLA-A2^neg^ SupT1 cells (S1 Fig) expressing SCT-M1 were incubated with DMF5 mCherry-His-sTCR (Fig 4b, red), and subsequently placed on ice to stop endocytosis. Anti-His antibodies were added on ice to distinguish surface bound from internalized sTCR (Fig 4b). Co-localization of the His-tag and the mCherry was detected on the surface, whereas punctuated intracellular structures were positive for mCherry only, indicating that the mCherry-TCR had been internalized (Fig 4b). We next showed that nanobeads coated with DMF5 sTCR were endocytosed by target cells expressing SCT-M1 (Fig 4c), and that sTCR coated onto the larger Dynabeads (2.8 μm in diameter) selectively formed rosettes with target cells expressing SCT-M1 (S2 Fig). Collectively, these results demonstrated that not only can the sTCR be internalized by itself, but also when it is fused to a protein (mCherry, 28kD) or bound to nano-sized beads. It is therefore likely that sTCRs could be exploited to efficiently transport molecules inside cells. Furthermore, our results indicated that sTCRs coupled to MACS or Dynabeads could be utilized to separate cells in a mixed population.

### MART-1 sTCR conjugated to toxins can specifically eliminate target cells

**Fig 5:**
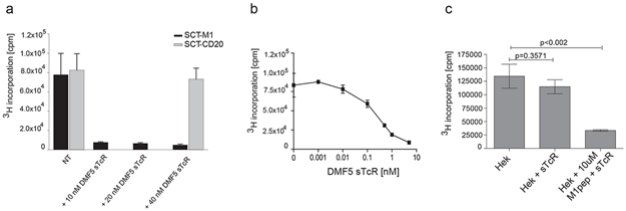
Antigen-specific elimination of target cells by sTCR-Saporin as measured by ^3^H-thymidine incorporation. (a) SupT1 cells constitutively expressing SCT-M1 or SCT-CD20 were incubated with or without (NT) 10–40 nM DMF5 sTCR-Sap for 3 days. Cells were then labeled with ^3^H-thymidine and radioactive incorporation was measured (cpm). (b) SupT1 cells expressing SCT-M1 were incubated with increasing amounts of DMF5 sTCR-Sap for 3 days and ^3^H-thymidine incorporation was plotted as in (a). (c) HEK293 cells were pulsed with or without 10 μM MART-1p in the presence or absence of 20 nM DMF5 sTCR-Sap for 3 days, and ^3^H-thymidine incorporation was determined. The experiments shown in (a-c) are each representative of two performed and error bars represent SD from triplicates.

Saporin is a 30kD plant toxin that upon ER to cytosol translocation inactivates ribosomes[38]. The SA-Sap lacks a targeting domain and is thus unable to enter into mammalian cells by itself. We bound biotinylated MART-1-sTCR to streptavidin-conjugated saporin (SA-Sap). Even at low concentrations of sTCR-Sap, a complete block in thymidine incorporation in the SCT-M1 expressing cells was observed (Fig 5a, black bars). The specificity of the Saporin delivery was confirmed, as SCT-CD20 expressing cells treated with the highest dose of DMF5 sTCR-Sap were resistant (Fig 5a, grey bars). We then performed kinetics and found that 0.1 nM sTCR-Sap was sufficient to reduce proliferation of the SCT-M1 expressing cells by approximately 50% (Fig 5b).

Similar results were obtained using HLA-A2^pos^, MART-1^neg^ HEK293 cells incubated with DMF5 sTCR-Sap, to which the cells were sensitive only when pulsed with MART-1p (Fig 5c).

**Fig 6:**
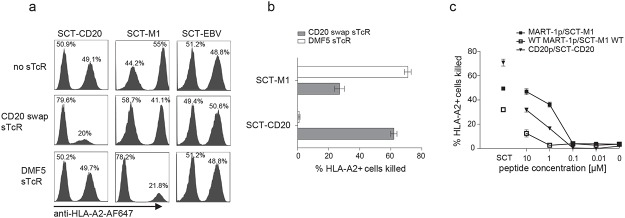
Antigen-specific elimination of target cells by sTCR-Saporin as measured by flow cytometry. (a) SupT1 cells expressing the indicated SCT were mixed at a 1:1 ratio with HLA-A2^neg^ SupT1 cells and co-cultured for 3 days in the presence of relevant or irrelevant sTCR conjugated to Saporin, as indicated. At end of culture, cells were stained with anti-HLA-A2 AF647 to determine the frequencies of HLA-A2 positive and negative target cells among FSC^hi^,SSC^hi^ events using flow cytometry. (b) Assay was performed as described in (a) and the percentage of SupT1 cells expressing indicated SCT that was eliminated by addition of sTCR is shown, calculated as described in materials and methods. Error bars indicate SEM of n = 3. (c) HLA-A2^pos^ SupT1 cells mixed at a ratio of 1:1 with HLA-A2^neg^ SupT1 cells were incubated overnight with indicated concentrations of peptide, washed, and indicated sTCR-Sap was added. SupT1 cells expressing SCT were used as positive controls (disconnected symbols). Culture and analysis to determine the percentage of eliminated HLA-A2^pos^ targets was performed as described in (a,b). Results shown are representative of two experiments performed, and error bars represent SD of duplicates.

Next, we used an alternative, flow cytometry-based, assay to study the ability of the sTCRs to specifically eliminate target cells. The MART-1 sTCR-Sap eliminated on average 71% (SEM = 2.24, n = 3) and the CD20 swap sTCR-Sap on average 62% (SEM 2.04, n = 3) of relevant target cells (Fig 6a). Vice versa, the MART-1 sTCR-Sap did not eliminate target cells expressing the SCT-CD20, whereas the artificially constructed CD20 swap sTCR-Sap reduced the frequency of SCT-M1 by 27%, indicating some cross-reactivity (Fig 5d and 6a). However, this sTCR did, similarly to the MART-1 sTCR, not affect the survival of SCT-EBV expressing target cells (Fig 5d). The MART-1 sTCR-Sap was able to target cells loaded with 1 μM of the MART-1p or with 10 μM of the lower affinity WT MART-1p. A similar difference was seen for the corresponding SCT, respectively. The CD20 swap sTCR-Sap could eliminate target cells pulsed with 1 μM CD20p (Fig 6c). The MART-1 sTCR-Sap did not affect target cells loaded with high concentrations of the CD20p. The peptide concentrations needed for elimination of target cells matched those needed to detect positive staining, shown in Fig 3c.

### Production of additional sTCRs

Finally, the sTCR production method was tested for two additional TCRs. A TCR specific for the HLA-A2/CD20p complex (A94) [33] was shown to be CD8 co-receptor-independent (S3a Fig) and stain its cognate pHLA target, but only when tetramerized (S3b Fig). The A94 sTCR-Sap specifically killed SupT1 cells expressing HLA-A2 and loaded with CD20p (S3c Fig). Finally, a sTCR recognizing an EBV-derived peptide (GLCTLVAML) specifically stained SCT-EBV-transduced SupT1 cells (S3d Fig). Thus, in total four functional sTCRs were produced by the novel approach introduced in this paper.

## Discussion

In the present study, successful production of four functional soluble TCRs capable of recognizing their cognate peptide-HLA ligands was achieved by the use of a simplified novel approach for cloning and expression in human cells. The transmembrane and intracellular domains of both TCR chains were removed and their coding sequences linked by a ribosomal skipping sequence 2A from picorna virus [22]. It is now accepted that the use of this sequence represents the most efficient way to generate equimolar amounts of a multimeric protein product [21] as compared with linkage using an internal ribosomal entry site sequence, or by use of two-plasmid transfection [39]. Indeed, unlike what has been reported for the production in baculovirus [40], we found that several sTCR were readily generated without the aid of artificial dimerization motifs. However, by modifying the chains with addition of a cysteine bridge to the C-domains or leucine zippers, sTCRs could also be efficiently produced as heterodimers from chains that were otherwise not possible to pair, as shown here for the CD20swap sTCR. Similar modifications have previously been shown to stabilize full-length TCRs [34, 41, 42] or bacterially produced sTCR [3, 7, 8, 35, 36]. However, it remains to be seen if the modifications can overcome production difficulties for all sTCR, making this mammalian platform universal. Importantly, the addition of a cysteine bridge or a leucine zipper could have an impact on the folding of the sTcR, although the results presented here for two different sTCRs show that the specificity was kept following these modifications. Since the recognition of the pHLA complex is conformational, this suggests that the overall structure of the TCR was maintained. Finally we have not scaled up the production of our sTcR for crystallographic analysis, but it would be interesting to compare such a product with bacterially produced sTcRs refolded from inclusion bodies.

Indeed, the supernatant from the sTCR-producing cells could be directly used for detection of the cognate pHLA complex by two of the sTCRs. This implies that advanced expression facilities and biochemical manipulations, such as refolding, can be avoided. Addition of various tags to the sTCR chains facilitated successful purification, labeling, or combination with desired cargo. The sTCR selectively bound its cognate pHLA complex both as a monomer and multimerized to SA, or attached to SA-conjugated nanobeads or large SA-Dynabeads. As expected, multimerization yielded an increase in the staining intensity of cells as compared to the monomer. Interestingly, in addition to labeling surface pHLA complexes, sTCRs were also endocytosed into target cells while carrying large cargo, such as the 28 kD mCherry. By coupling the active subunit of the toxin Saporin (30 kD) to the sTCRs, we moreover demonstrated that the cargo was not only kept in intracellular vesicles, but further retrotranslocated to the cytosol. The results open new avenues for the use of sTCR as therapeutic agents for specific killing of selected antigen-expressing cells or for the detection or isolation of altered or infected cells.

In contrast to antibodies that detect molecules present on the cell surface, TCRs are capable of recognizing any protein in the context of MHC. This is a substantial advantage over antibodies: any disease marker hidden within the cell can potentially be targeted by a TCR. Recent success stories have shown the power of therapeutic monoclonal antibodies such as Rituximab and Herceptin [43]. It is therefore expected that the development of sTCRs as carrier agents will enlarge the spectrum of specific drug targeting, as shown using the single chain TCR [18]. Furthermore, sTCR could in principle be exploited in diagnostics to discover the targets of TCRs derived from T cells involved in disease. However, for natural TCRs the relatively low affinity for the pMHC complex represents a limitation. Modifications, such as in vitro affinity maturation [44–47], multimerization of sTCRs and improved stabilization of the dimer, as shown for the CD20 swap sTCR, can synergize to increase the affinity of the sTCR for the pHLA complex and thus the preparation of molecules that would complement antibodies. Affinity maturation can, however, substantially change the reactivity of the natural TCR. Therefore, the recent development of technology that allows the use of non-affinity matured soluble TCRs for discovery of naturally occurring TCR peptide-ligands[23] represents a major advance that could lead to the identification of the driving antigen in T cell-mediated autoimmune disease as well as in curative immune responses to cancer. For this purpose, the currently presented method for production of sTCR that are minimally modified relative to their natural counterparts, might be particularly advantageous.

Collectively, we believe that the main value of our technology lies in its ease of use and potential to increase the availability of sTCRs for further studies by a multitude of research groups without special competence in advanced protein production and purification. This should facilitate further technological progress to overcome challenges, including the inherently low affinity of TCRs for antigen, leading to increased applicability of sTCRs. In this regard, it is interesting to note that the humanization of monoclonal antibodies has been essential to reduce immunogenicity and thereby widen therapeutic applications [48]. Similarly, it is expected that the production of sTCRs that are minimally modified relative to their full-length counterparts, and equipped with the posttranslational modifications introduced in human cells, will further facilitate the therapeutic use of these reagents.

## Supporting Information

S1 Fig Cells modified to express HLA-A2 SCT stain positively with anti-HLA-A2. SupT1 cells, which are HLA-A2 negative, were transduced withSCT-M1, SCT-CD20 or SCT-EBV constructs, respectively. Cells staining positively with anti-HLA-A2 were sorted, expanded and stained with anti-HLA-A2 antibodies conjugated to Alexa Fluor 647 (anti-HLA-A2-AF647), as shown. Untransduced SupT1 cells (filled grey) were used as a negative control.
(TIF)

S2 Fig Antigen-expressing target cells form rosettes when mixed with sTCR-coated Dynabeads. SupT1 cells expressing SCT-M1 or SCT-irr fused to GFP were mixed and stained with DMF5 sTcR-mCherry conjugated to SADynabeads at 37°C. Clusters of cells were analyzed by direct fluorescence microscopy.
(TIF)

S3 Fig The A94 sTCR is CD8 co-receptor independent and can specifically kill target cells expressing HLA-A2/CD20p when conjugated to Saporin. (a) J76 CD8 positive cells were transduced to express the full-length HLAA2/CD20p-specific TCR A94 (black bars) or A64 (grey bars). The cells were cocultured with SupT1 cells expressing either wild type HLA-A2 or the mutant HLAA2 DT227/228KA (HLA-A2-DK); the latter is unable to bind the CD8 co-receptor. The SupT1 cells were pre-loaded or not with 10 μM of CD20p, as indicated. After 24 hours, the cell supernatants were harvested and levels of IL-2 measured by ELISA. Error bars represent the standard deviation (SD) of duplicates in one experiment and the experiment was repeated once. (b) SupT1 cells expressing SCT-CD20 were incubated with A94 sTcR-SA-AF647 and analyzed by flow cytometry (bold line). Staining of SupT1 cells expressing SCT-EBV was used as a negative control (filled grey). (c) SupT1 cells constitutively expressing HLA-A2 were loaded with 10 μM of the indicated peptide (CD20p, MART-1p or EBVp) 12 hours prior to the addition of either the A94 or the DMF5 sTCRs conjugated to the toxin Saporin, and were cultured for 3 days. Cells were then labeled with 3H-thymidine and radioactive incorporation was counted (cpm). Error bars represent SD from triplicates and the experiment was performed twice with A94. (d) SupT1 cells expressing SCT-EBV were incubated with the EBV-specific sTCR-SA-647 and analyzed by flow cytometry (bold line). Staining of HLA-A2 transduced SupT1 cells was used as a negative control (filled grey).
(TIF)
