# Supplementary figures and images for: Soluble T-Cell Receptors Produced in Human Cells for Targeted Delivery
Source: PLoS One. 2015 Apr 13;10(4):e0119559. doi: 10.1371/journal.pone.0119559 (PMC4395278; doi:10.1371/journal.pone.0119559)

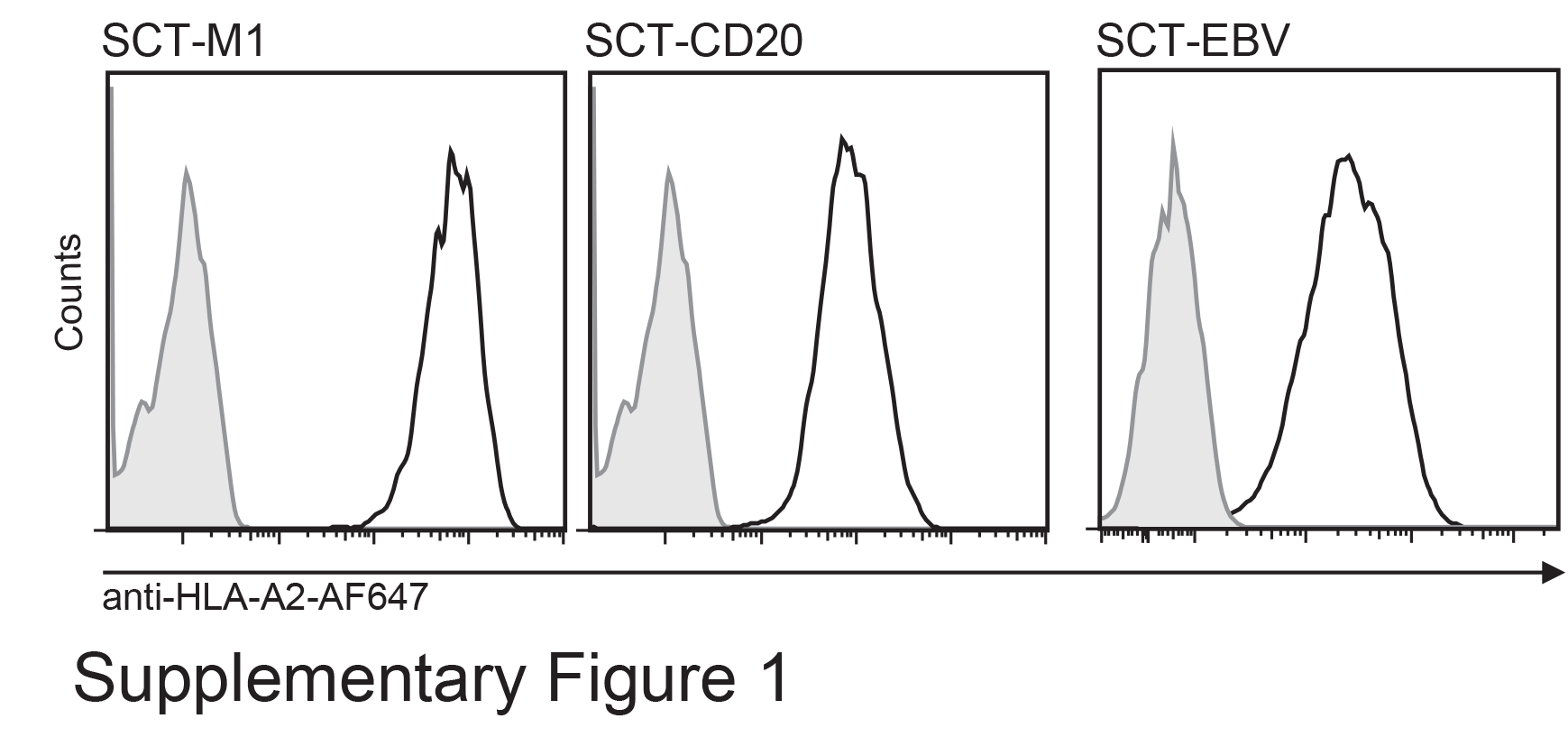

Supplement: S1 Fig — SupT1 cells, which are HLA-A2 negative, were transduced withSCT-M1, SCT-CD20 or SCT-EBV constructs, respectively. Cells staining positively with anti-HLA-A2 were sorted, expanded and stained with anti-HLA-A2 antibodies conjugated to Alexa Fluor 647 (anti-HLA-A2-AF647), as shown. Untransduced SupT1 cells (filled grey) were used as a negative control. (TIF) [file pone.0119559.s001.tif]

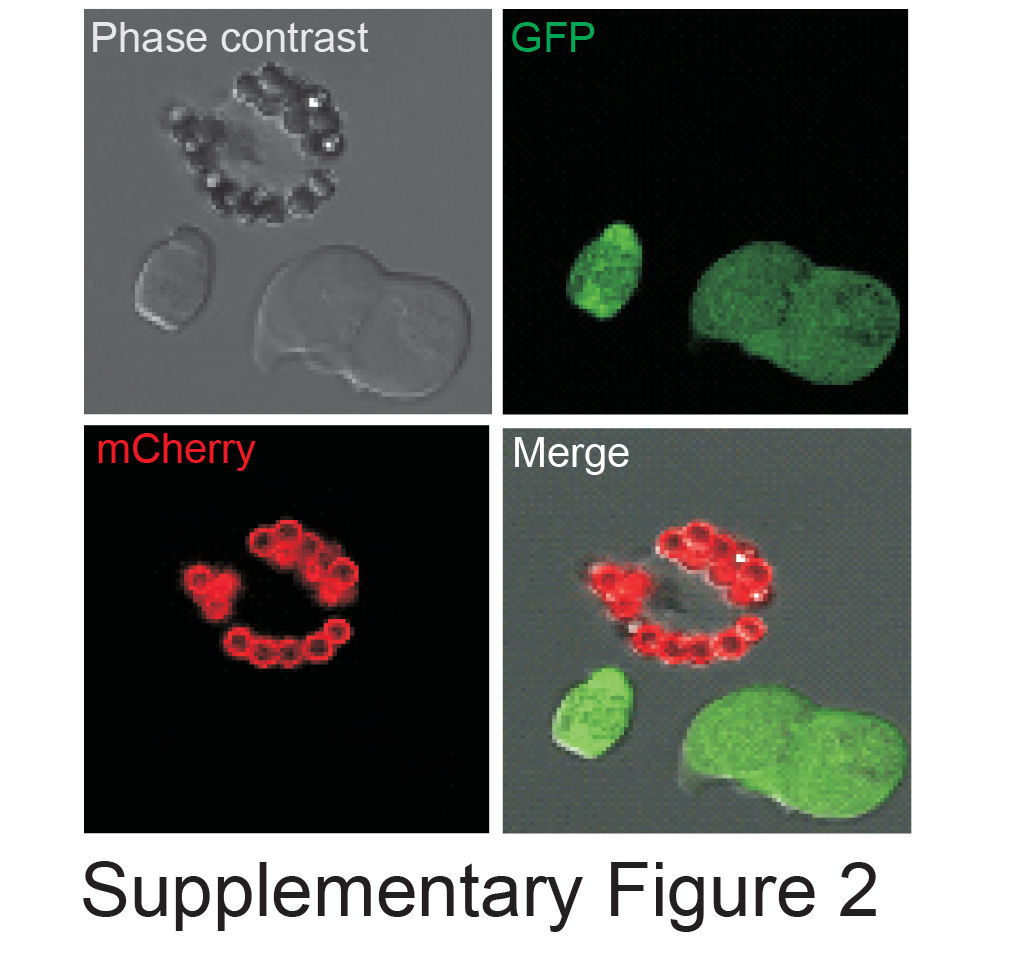

Supplement: S2 Fig — SupT1 cells expressing SCT-M1 or SCT-irr fused to GFP were mixed and stained with DMF5 sTcR-mCherry conjugated to SADynabeads at 37°C. Clusters of cells were analyzed by direct fluorescence microscopy. (TIF) [file pone.0119559.s002.tif]

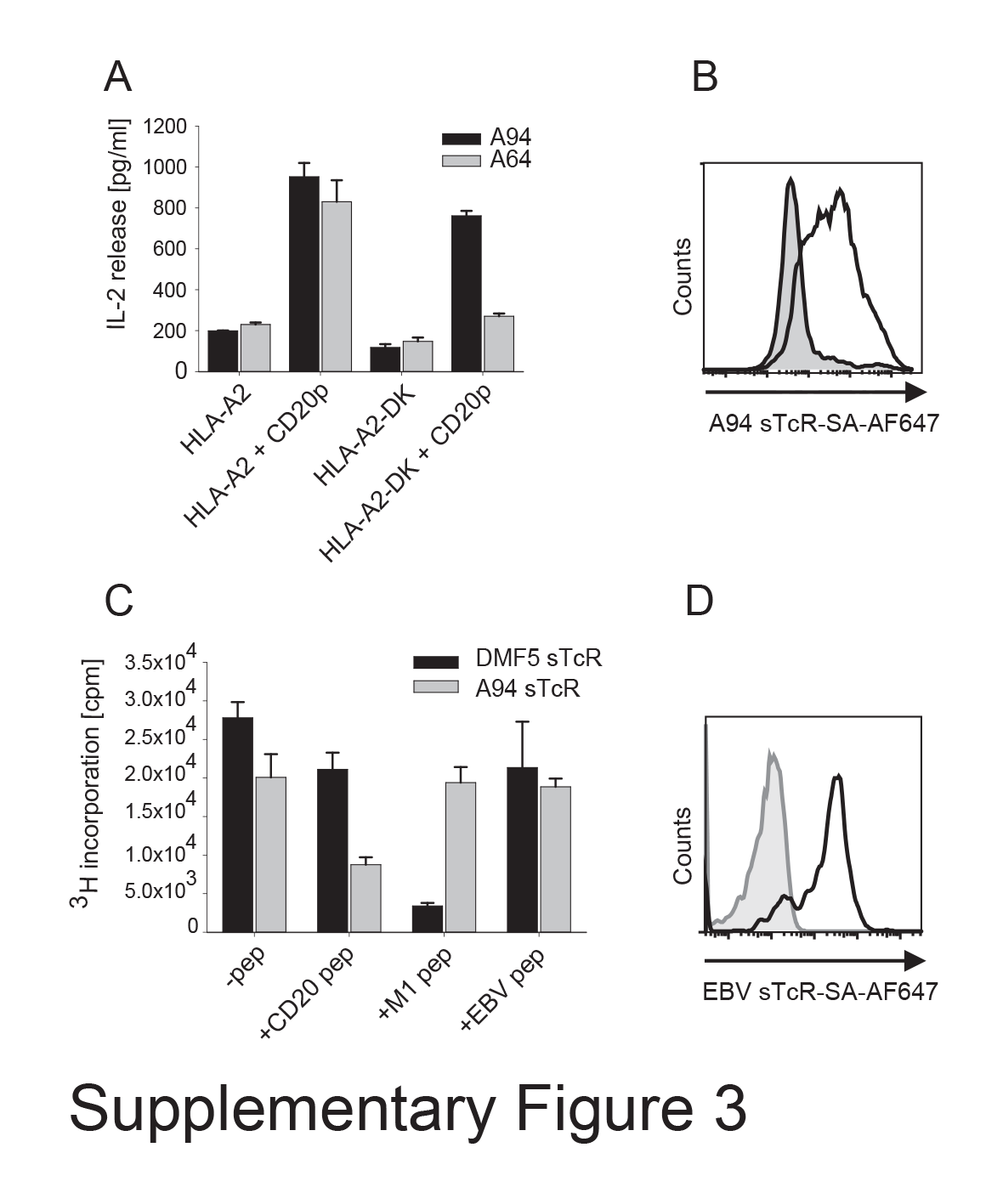

Supplement: S3 Fig — (a) J76 CD8 positive cells were transduced to express the full-length HLAA2/CD20p-specific TCR A94 (black bars) or A64 (grey bars). The cells were cocultured with SupT1 cells expressing either wild type HLA-A2 or the mutant HLAA2 DT227/228KA (HLA-A2-DK); the latter is unable to bind the CD8 co-receptor. The SupT1 cells were pre-loaded or not with 10 μM of CD20p, as indicated. After 24 hours, the cell supernatants were harvested and levels of IL-2 measured by ELISA. Error bars represent the standard deviation (SD) of duplicates in one experiment and the experiment was repeated once. (b) SupT1 cells expressing SCT-CD20 were incubated with A94 sTcR-SA-AF647 and analyzed by flow cytometry (bold line). Staining of SupT1 cells expressing SCT-EBV was used as a negative control (filled grey). (c) SupT1 cells constitutively expressing HLA-A2 were loaded with 10 μM of the indicated peptide (CD20p, MART-1p or EBVp) 12 hours prior to the addition of either the A94 or the DMF5 sTCRs conjugated to the toxin Saporin, and were cultured for 3 days. Cells were then labeled with 3H-thymidine and radioactive incorporation was counted (cpm). Error bars represent SD from triplicates and the experiment was performed twice with A94. (d) SupT1 cells expressing SCT-EBV were incubated with the EBV-specific sTCR-SA-647 and analyzed by flow cytometry (bold line). Staining of HLA-A2 transduced SupT1 cells was used as a negative control (filled grey). (TIF) [file pone.0119559.s003.tif]
